# Pholidonone, an active stilbene derivative from *Pholidota cantonensis*, exhibits pro-apoptotic effect *via* induction of endoplasmic reticulum stress in human gastric cancer

**DOI:** 10.29219/fnr.v63.3553

**Published:** 2019-09-06

**Authors:** Liang Liu, Wei Wang, Zhichen Zhao, Chen Hu, Li Tao, Xianwen Zhang

**Affiliations:** 1 Institute of Translational Medicine, Medical College, Yangzhou University, Yangzhou, China; 2 The Key Laboratory of Syndrome Differentiation and Treatment of Gastric Cancer of the State Administration of Traditional Chinese Medicine, Yangzhou University, Yangzhou, China; 3 Jiangsu Key Laboratory of Zoonosis, Jiangsu Co-innovation Center for Prevention and Control of Important Animal Infectious Diseases and Zoonoses, College of Veterinary Medicine, Yangzhou University, Yangzhou, China; 4 Oncology Department, Clinical Medical College, Yangzhou University, Subei People’s Hospital of Jiangsu Province, Yangzhou, China

**Keywords:** Pholidota cantonensis, pholidonone, ER stress, apoptosis, CHOP

## Abstract

**Background:**

Gastric cancer (GC) is the second leading cause of cancer death worldwide. Current chemotherapeutic drugs exert therapeutic effects accompanied by severe side effects. Therefore, it is imperative to urgently find new drugs with low toxicity and high efficacy for the treatment of GC. Natural products as well as functional foods have always been rich sources of potential antitumor agents. *Pholidota cantonensis* Rolfe, a well-known functional food and a traditional Chinese medicine, has been used for a long time in China for inflammatory diseases. Previously, we have evaluated its possible antitumor potentials by screening different solvent extracts, and found that the ethyl acetate (EtOAc) extract showed potent cytotoxicity on human GC cell line AGS with an IC_50_ value of 33.68 ± 1.68 μg/mL. In view of the poor knowledge concerning the phytochemical and pharmacological study of *P. cantonensis*, it is essential to characterize the active compounds from EtOAc extract and the mechanisms of action underlying the antitumor effect of the herb.

**Objective:**

This study aimed to identify the primary compounds in EtOAc extract of *P. cantonensis* involved in the antitumor activity of the plant by evaluating the cytotoxicity in two human GC cell lines, including AGS and BGC-823 cells. Since endoplasmic reticulum (ER) stress-induced cell apoptosis represents attractive targets for cancer therapy recently, we focused on the underlying mechanisms associated with ER stress-induced cell apoptosis and related signaling pathways.

**Methods:**

Various chromatographic techniques, including silica gel, Sephadex LH-20, and octadecylsilyl silica gel (ODS) C_18_, were used to separate the main active compound from EtOAc extract of *P. cantonensis*. The cell viability of AGS and BGC-823 cells upon purified compound treatment was determined by a 3-(4,5-dimethyl-2-thiazolyl)-2,5-diphenyl-2H-tetrazolium bromide (MTT) assay. The alteration of cell morphology was observed using an inverted microscope. Cell apoptosis was determined by fluorescein isothiocyanate (FITC)-labeled annexin-V/propidium iodide (PI) double-staining and flow cytometry analysis. Western blot analyses were performed to examine the levels of intracellular signaling molecules involved in ER stress-induced apoptosis.

**Results:**

A rare stilbene derivative pholidonone was isolated and identified. The results showed that pholidonone displayed potent cytotoxicity on human GC cells. The IC_50_ values for 24 and 48 h in AGS cells were 26.54 ± 0.32 and 25.20 ± 3.67 μM, and the IC_50_ values for 24 and 48 h in BGC-823 cells were 32.41 ± 3.83 and 17.28 ± 2.30 μM, respectively. In addition, pholidonone had pro-apoptotic effect on AGS and BGC-823 cells, and it upregulated the levels of proteins involved in ER stress, including BiP, PDI, Calnexin, Ero1-Lα, IRE1α, PERK, CHOP, and cleaved-caspase-3 in AGS and BGC-823 cells.

**Conclusion:**

Pholidonone can trigger ER stress-induced apoptosis through PERK and IRE1α signaling pathways. Pholidonone might be a potential naturally occurring antitumor agent.

## Popular scientific summary

- Pholidonone is a stilbene derivative with prominent antitumor activity, which is isolated from ethyl acetate extract of *Pholidota cantonensis* Rolfe, a well-known functional food and traditional Chinese medicine.
- The present study indicates that pholidonone can trigger apoptotic cell death in human gastric cancer cells, which is attained by induction of ER stress probably *via* PERK and IRE1α signaling pathways.
- As a functional food ingredient, pholidonone might be a promising candidate drug for the treatment of cancer in the future.

According to the American Cancer Society, gastric cancer (GC) is the second leading cause of cancer death globally (1). At present, surgery remains the most effective therapeutic strategy with the highest response rate for GC. However, patients with advanced GC are frequently diagnosed with unresectable disease (2). For advanced GC, chemotherapy is the main mode of treatment (2). However, the first-line chemotherapeutic drugs, such as fluorouracil, cisplatin, and taxane, have therapeutic effects accompanied by severe side effects. Therefore, it is essential to find new drugs with low toxicity and high efficacy for the treatment of GC.

*Pholidota cantonensis* Rolfe belongs to the Orchidaceae family and is widely distributed in China. The whole plant or pseudobulb of *P. cantonensis*, named ‘Xiaoshixiantao’, has been a famous functional food, especially in the southern regions of China (3). It is used to be stewed with various meats to prepare delicious and nutritious medicinal dishes that are beneficial for human health. *P. cantonensis* is a kind of heat-clearing and detoxifying traditional Chinese medicine (TCM), and this kind of TCM has a long history of being used by practitioners of Chinese medicine to treat tumors. Modern pharmacological investigations have showed that *P. cantonensis* has antitumor, anti-inflammatory, and antioxidant effects (3–5). Meanwhile, previous phytochemical investigations have revealed that stilbenes and phenanthrenes are the major constituents of *P. cantonensis* (4–8). However, relevant phytochemical and pharmacological studies of this plant are poorly understood, which limits its further development and use.

Edible medicinal plants, with therapeutic effects and high safety, have always been sources of new antitumor drugs. In the course of our screening for edible medicinal plants with antitumor properties, ethyl acetate (EtOAc) extract of *P. cantonensis* caught our attention, as it was found to display potent cytotoxicity on human GC AGS cells with an IC_50_ value of 33.68 ± 1.68 μg/mL *in vitro*. Therefore, chromatographic techniques were used to separate the main active compounds from this extract, and the specific mechanisms and underlying signaling pathways associated with endoplasmic reticulum (ER) stress-induced apoptosis were also elucidated.

## Materials and methods

### Materials

The aerial part of *P. Cantonensis* was collected in Lishui, Zhejiang Province of China in March 2014. The plant materials were identified by Prof. Hu-Yin Huai, School of Biological Science and Technology of Yangzhou University, and a voucher specimen (No.XYSXT20140328) was deposited at the Herbarium of Pharmacy Department, Medical College of Yangzhou University. Cisplatin (cis-diaminedichloroplatinum, DDP) injection was purchased from Nanjing Pharmaceutical Co., Ltd. (1 mg/mL) and diluted with phosphate buffer saline (PBS). Tunicamycin (TM, >98%) powder and all the antibodies used in western blot analyses were purchased from Cell Signaling Technology Company (Danvers, MA, USA).

### Preparation of pholidonone

The powdered, dry whole grass of *P. Cantonensis* (15 kg) was extracted four times with 95% EtOH at 85°C, each extraction lasting 2 h. The extract was concentrated at reduced pressure, and then partitioned successively with EtOAc and *n*-BuOH. The EtOAc part of *P. cantonensis* was found to display potent cytotoxicity to human GC AGS cells with an IC_50_ value of 33.68 ± 1.68 μg/mL *in vitro*, which was then separated by column chromatography using silica gel, sephadex LH-20, and ODS C_18_ to obtain pholidonone. Pholidonone was dissolved in dimethylsulfoxide (DMSO) as 100 mM stock solution.

### Cell line and cell culture

Human GC AGS and BGC-823 cell lines were purchased from Cell Bank of Shanghai Institutes for Biological Sciences of Chinese Academy of Sciences. Cells were cultured in RPMI-1640 medium supplemented with 10% fetal bovine serum (FBS), 100 U/mL penicillin, and 100 μg/mL streptomycin. Cultures were maintained in a humidified environment with 5% CO_2_ at 37°C.

### Cell morphology assessment

AGS and BGC-823 cells were cultured in RPMI-1640 medium at the mid-logphase for experimental use. DMSO (as the control in all experiments, final concentration of 0.5%) or pholidonone (final concentration of 5, 10, 20, 40 μM) were added into the culture medium. DDP with a final concentration of 10 μg/mL was used as the positive control. After incubation for 48 h, images of the cell morphology were taken with an inverted microscope (Olympus IX-70).

### Cell viability assay

The effect of pholidonone on the cell viability of AGS and BGC-823 cells was determined by an MTT assay. Briefly, AGS and BGC-823 cells at the mid-log phase were seeded in a 96-well plate at a density of 5 × 10^3^ cells per well in 200 μL medium. After being cultured overnight, cells were exposed to 0, 12.5, 25, 50, 100, or 200 μM pholidonone for 24 and 48 h, respectively. DDP with a final concentration of 10 μg/mL was used as the positive control. Then, MTT (final concentration was 454.5 μg/mL) was added, and the cells were incubated for another 4 h at 37°C. The medium was abandoned 4 h later, and DMSO (150 μL) was added. The spectrophotometric absorbance at 490 nm was measured by EnSpire^TM^ Multilabel Reader (PerkinElmer). The concentration of pholidonone resulting in 50% inhibition of control growth (IC_50_) was calculated *via* SPSS Statistics 16.0 for Windows.

### Apoptosis analysis by flow cytometry

Cell apoptosis was determined by FITC-labeled annexin-V/PI double-staining and flow cytometry analysis. Briefly, AGS and BGC-823 cells were treated with 0, 5, 10, 20, and 40 μM pholidonone for 48 h. At the indicated time, cells were harvested, and apoptosis detection was performed by the FITC-annexin V apoptosis detection kit (KeyGEN) according to the manufacturerʼs protocol. Percentages of necrotic cells (in the upper left quadrant), living cells (in the lower left quadrant), and apoptotic cells (in the upper right and lower right quadrants for ‘late’ and ‘early’ apoptotic cells, respectively) were determined by flow cytometry (FACSAria SORP; Becton Dickinson).

### Western blot analysis

AGS and BGC-823 cells were cultured in RPMI 1640 medium at the mid-log phase and then incubated with pholidonone at 0, 5, 10, 20, or 40 μM for 48 h. After the treatments, cells were washed by pre-cooled PBS and lysed in RIPA buffer supplemented with 1mM PMSF and protease inhibitor cocktail (Bestbio) at a dilution of 1:100. Protein concentration was determined using the BCA^TM^ protein assay kit (Pierce). Protein samples (50 μg) were loaded on SDS-PAGE and then transferred to a PVDF membrane (Millipore). After blocked with 5% nonfat dry milk in TBST for 2 h, the membranes were incubated with primary antibodies (1:1000, CST, including anti-BiP, anti-Calnexin, anti-Ero1-Lα, anti-IRE1α, anti-CHOP, anti-PERK, anti-PDI, and anti-cleaved-caspase-3) overnight at 4°C. The blots were washed and incubated with HRP-linked secondary antibodies (1:2000, CST) for 2 h at room temperature. Membranes were again washed three times with TBST and visualized using enhanced chemiluminescence substrate (Immobilon^TM^ Western; Millipore). TM was used as a positive control.

### Statistical analysis

All results are presented as means ± SD for three independent experiments. Differences between control and pholidonone treatments were performed by one-way analysis of variance (ANOVA). Differences were considered significant at *p* < 0.05.

## Results

### One rare stilbene derivative pholidonone is obtained from the active extract

**Fig. 1:**
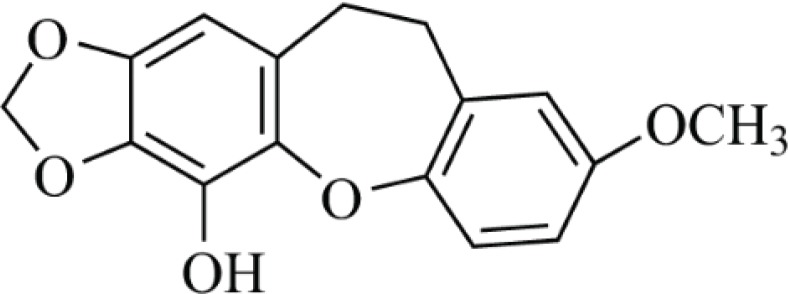
Chemical structure of pholidonone.

The purified compound was a white-like powder, EI-MS m/z 286[M]^+^; ^1^H NMR (600 MHz, CD_3_OD) *δ*: 6.29 (1H, s, H-1), 6.92 (1H, d, *J* = 8.4 Hz, H-5), 6.53 (1H, dd, *J* = 3.0, 8.4 Hz, H-6), 6.54 (1H, d, *J* = 3.0 Hz, H-8), 2.97 (4H, s, H-9, 10), 5.93 (2H, s, H-11), 3.79 (3H, s, OCH_3_); ^13^C NMR (150 MHz, CD_3_OD) *δ*: 109.1 (C-1), 140.2 (C-2), 135.7 (C-3), 154.7 (C-4), 127.9 (C-4a), 140.9 (C-4b), 122.3 (C-5), 114.5 (C-6), 151.1 (C-7), 117.4 (C-8), 133.7 (C-8a), 31.8 (C-9), 31.3 (C-10), 137.1 (C-10a), 102.8 (C-11), 57.2 (OCH_3_). The above spectra data were basically in agreement with literature (6). Therefore, it was identified as pholidonone (Fig. 1), which is a rare stilbene derivative with a seven-membered oxygen-containing ring and a methylenedioxy functionality.

### Pholidonone inhibits the cell viability in human GC cell lines

**Fig. 2:**
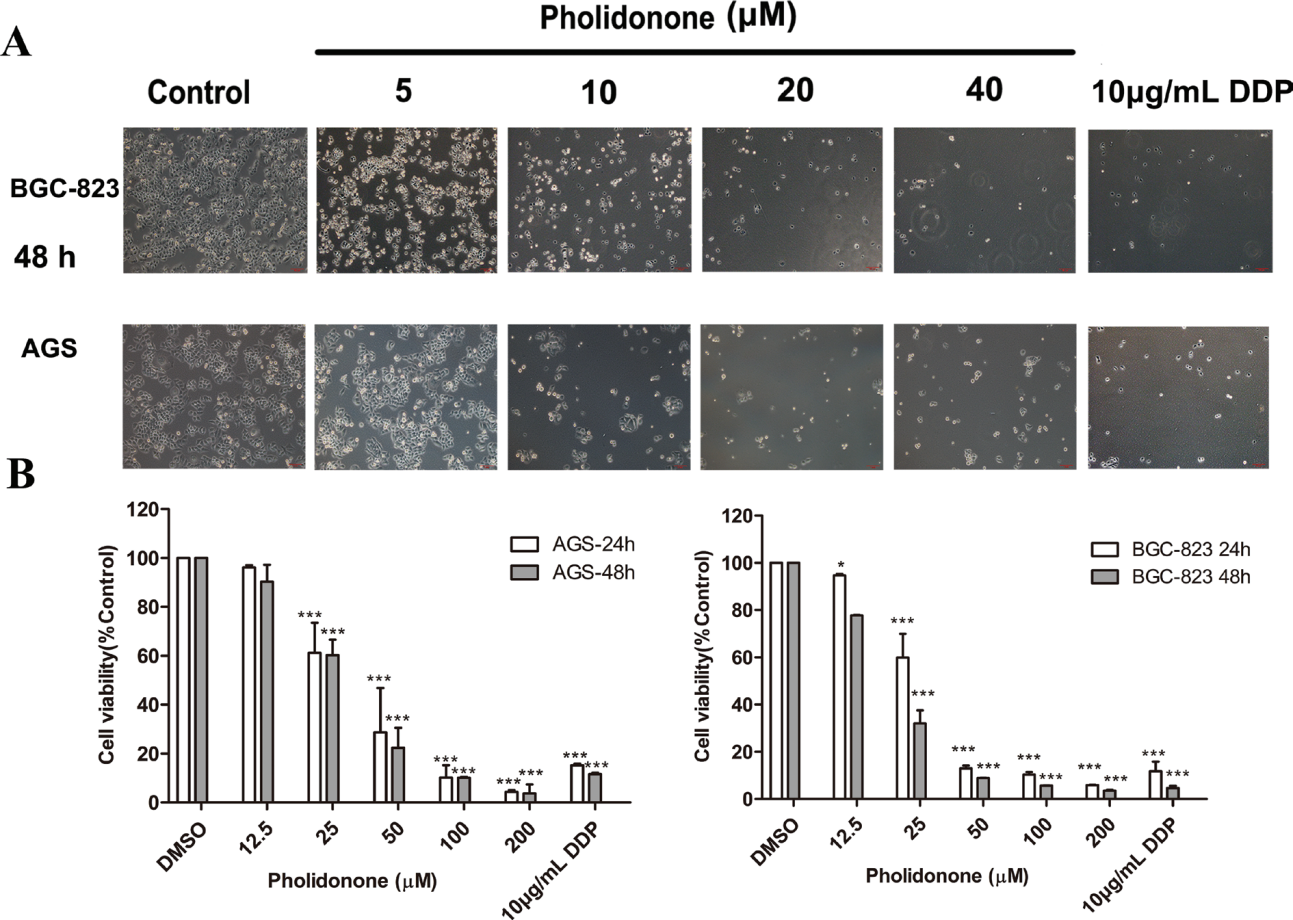
The inhibitory effect of pholidonone on AGS and BGC-823 cells viability. Morphological change of AGS and BGC-823 cells after incubation with pholidonone (0, 5, 10, 20, and 40 μM) for 48 h (magnification × 100) (A). The effect of pholidonone on the cell viability of AGS and BGC-823 cells (B). AGS and BGC-823 cells were treated with pholidonone (0, 12.5, 25, 50, 100, and 200 μM) for 24 and 48 h, respectively, and DDP at 10 μg/mL was used as a positive control. Data are presented as means ± SD by three independent experiments. Statistical significance: ^*^p < 0.05, ^***^p < 0.001, compared with control (DMSO).

To determine the anti-carcinogenic effect of pholidonone in an *in vitro* cell culture model, we first determined the effect of pholidonone on cell viability of cancer cells using an MTT assay. The results showed that pholidonone had significant inhibitory effect on the viability of AGS and BGC-823 cells in a time- and dose-dependent manner (Fig. 2B). First, the AGS and BGC-823 cells were treated with varying concentrations of pholidonone (0, 12.5, 25, 50, 100, and 200 μM) for 24 and 48 h, respectively. The IC_50_ values of pholidonone for 24 and 48 h in AGS were 26.54 ± 0.32 and 25.20 ± 3.67 μM, respectively, while the IC_50_ values in BGC-823 cells were 32.41 ± 3.83 and 17.28 ± 2.30 μM, respectively. Dramatic changes in cell morphology were also observed between pholidonone-treated and control cells (Fig. 2A). In pholidonone-treated cells, adherent and spindle-shaped AGS and BGC-823 cells were shrunk and transformed into semi-suspended and sphere-shaped cells with 10 μM of pholidonone exposure. Treatment with pholidonone at 20 μM yielded a comparable effect with that of DDP at 10 μg/mL. All the above findings suggested pholidonone could induce cytotoxic effect on human GC cells.

### Pholidonone induces apoptosis in human GC cell lines

**Fig. 3:**
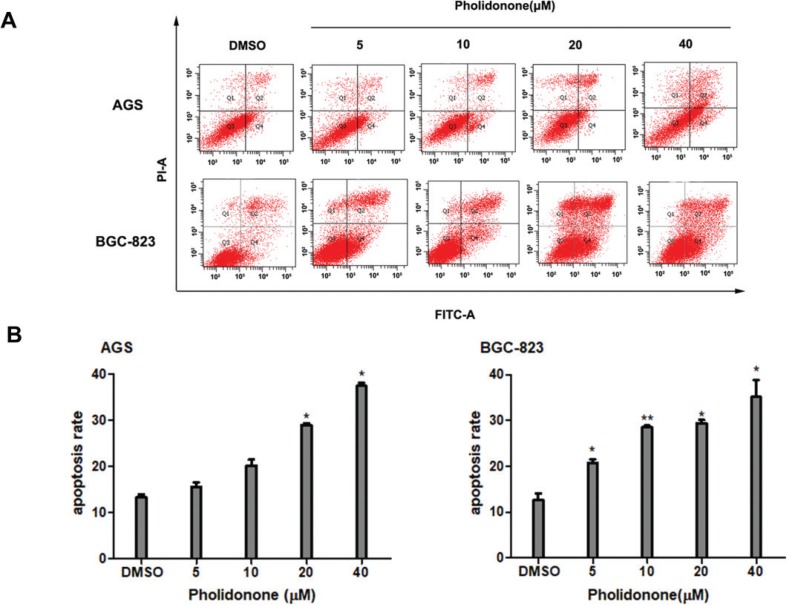
Pholidonone induces apoptosis in AGS and BGC-823 cells. Flow cytometric analyses of annexin V-FITC/PI double-staining in AGS and BGC-823 cells treated with pholidonone (0, 5, 10, 20, and 40 μM) for 48 h (A). The percentage of apoptotic cells in AGS and BGC-823 cells treated with pholidonone (0, 5, 10, 20, and 40 μM) for 48 h (B). Data are presented as means ± SD by three independent experiments. Statistical significance: ^*^p < 0.05, ^**^p < 0.01, compared with control (DMSO).

To examine whether pholidonone-induced cytotoxic effect on the human GC cells was associated with the induction of apoptosis, AGS and BGC823 cells were treated with various concentrations of pholidonone (0, 5, 10, 20, and 40 μM), and apoptosis was assessed using the FITC-labeled annexin V/PI staining and flow cytometry. Pholidonone-induced apoptotic cells were counted as ‘late’ or ‘early’ apoptotic cells, which were shown respectively in the upper right (UR) and lower right (LR) quadrants of the histograms. A dose-dependent increase in early apoptotic (annexin V^+^/PI^−^) cells in the presence of pholidonone was observed (Fig. 3A). The percentage of apoptotic cells (the total of ‘late’ and ‘early’ apoptotic cells) treated with 0, 5, 10, 20, and 40 μM of pholidonone was 13.73 ± 0.10%, 15.3 ± 1.25%, 20.07 ± 0.80%, 29.22 ± 0.80%, and 37.92 ± 1.70% in AGS cells and 12.7 ± 0.40%, 21.28 ± 0.50%, 28.84 ± 0.20%, 29.93 ± 3.25%, and 36.76 ± 0.50% in BGC-823 cells, respectively (Fig. 3B). These results demonstrated the pholidonone-induced morphological changes were characteristics of cell apoptotic death. Altogether, these findings provided strong evidence that pholidonone had pro-apoptotic effect on AGS and BGC-823 cells.

### Pholidonone triggers ER stress response in human GC cell lines

**Fig. 4:**
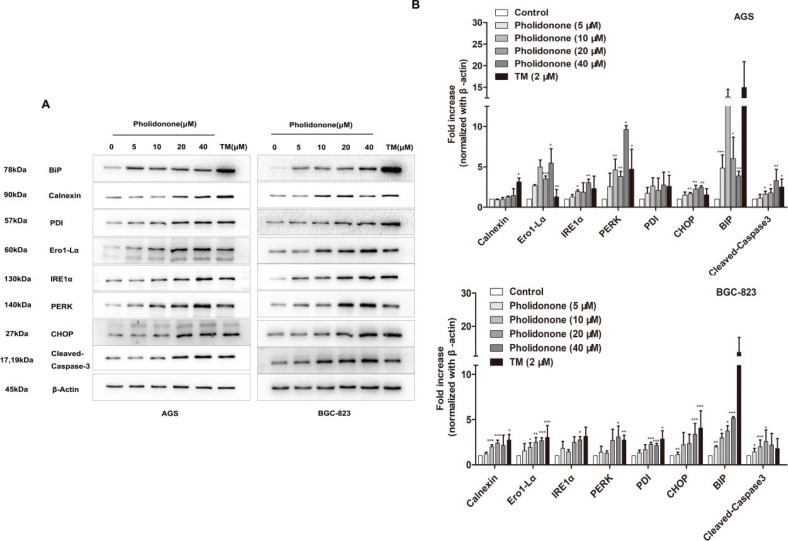
Pholidonone activates ER stress-related signals in AGS and BGC-823 cells. Protein expression levels of BiP, Calnexin, PDI, Ero1-Lα, IRE1α, PERK, CHOP, and Cleaved-caspase-3 in AGS and BGC-823 cells treated with 0, 5, 10, 20, and 40 μM pholidonone for 48 h, with β-actin as the control (A). Densitometry analysis of the protein levels of apoptosis-related proteins in AGS and BGC-823 cells treated with pholidonone (B). Data are presented as means ± SD by three independent experiments. Statistical significance: ^*^p < 0.05, ^**^p < 0.01,^***^p < 0.001, compared with control (DMSO).

Our aim was also to examine whether both cell lines follow a similar mechanism of apoptotic cell death after the treatment with pholidonone. As ER stress is a major contributor in apoptosis, it raises an important issue regarding the molecular links between ER stress and pholidonone-mediated pro-apoptotic effect on AGS and BGC-823 cells. ER-resident chaperones and folding enzymes, including BiP, PDI, Ero1-Lα, and IRE1α, are molecular markers of ER stress. Moreover, prolonged ER stress leads to PERK signaling-mediated upregulation of C/EBP homologous protein (CHOP), a pro-apoptotic transcription factor. Caspase-3 is a downstream caspase that is activated by each of the cell death pathways, and its active form (cleaved-caspase-3) is one of the key mediators of apoptosis in its execution phase. Thus, ER stress-apoptosis-interacting proteins were further studied by Western blot analyses. As a result, pholidonone significantly upregulated the protein expression levels of BiP, PDI, Calnexin, Ero1-Lα, IRE1α, PERK, CHOP, and cleaved-caspase-3 in AGS and BGC-823 cells (Figs. 4A and 4B). These findings indicated that pholidonone might induce AGS and BGC-823 cells apoptosis probably *via* PERK and IRE1α signaling pathways.

## Discussion

Currently, functional properties of plants, food, and human nutrition are potentially applied in cancer prevention and therapy (9). The increasing interest of consumers in functional foods and natural medicine has brought about a rise in demand for functional ingredients obtained using ‘natural’ processes. In the present study, an active stilbene derivative pholidonone was obtained from EtOAc extract of *P. cantonensis*. The effect of pholidonone on cell viability and cell apoptosis of human GC AGS and BGC-823 cells was investigated. Our data indicated that pholidonone had significant cytotoxicity on human GC cells. Flow cytometry analyses results suggested that the cytotoxic effect of pholidonone was mediated by inducing tumor cell apoptosis.

**Fig. 5:**
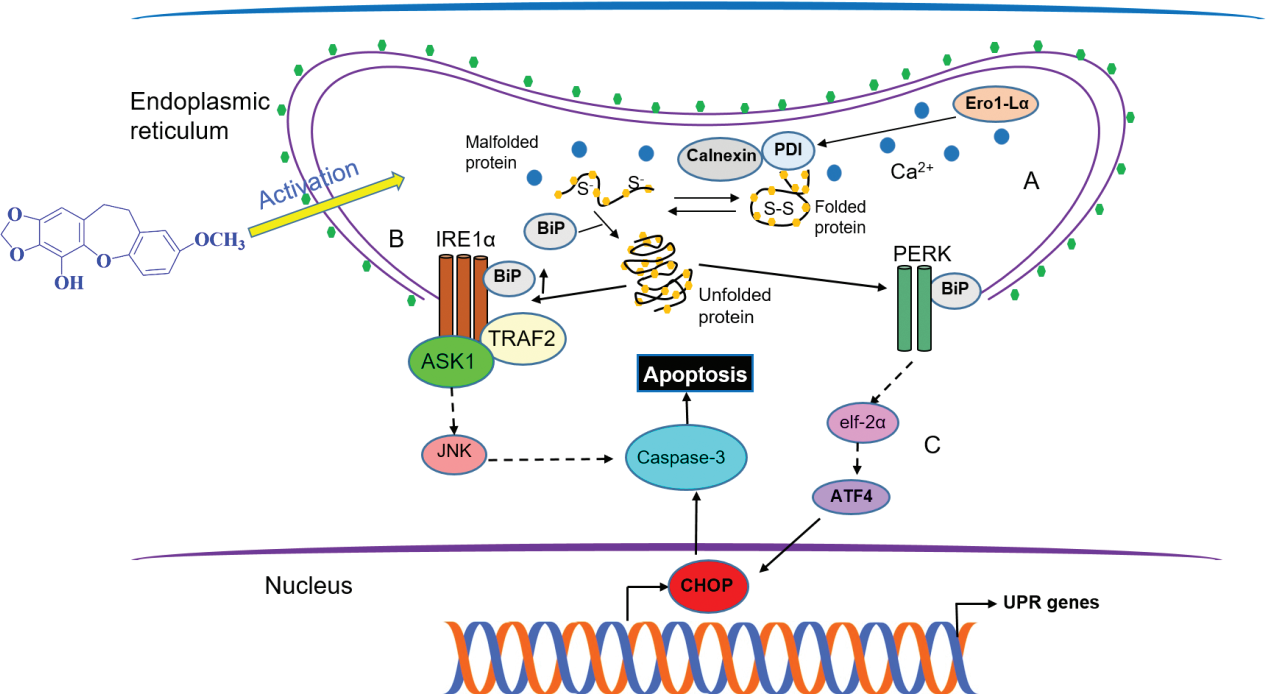
Proposed mechanisms of the pro-apoptotic effect on human GC AGS and BGC-823 cells induced by pholidonone. Increasing expression of Ero1-Lα, PDI, and calnexin was observed, suggesting that malfolded proteins were accumulated in the ER after pholidonone administration, causing ER stress and provoking the unfolded protein response to activate the signaling cascade that triggers apoptosis (A); Pholidonone might trigger cell apoptosis through upregulating IRE1α and subsequently activating Caspase-3 signaling *via* JNK (B); Pholidonone might exert its pro-apoptotic effect through the activation of CHOP *via* PERK signaling pathway, which subsequently leads to the upregulation of Caspase-3, eventually triggering apoptosis (C).

In order to understand the mechanism of action of pholidonone, the associated molecular signaling pathways were examined. ER is the cytoplasmic compartment where proteins and lipids are synthesized and modified. Proteins that do not mature properly are retained in the ER and eventually re-translocated to the cytosol for degradation by the 26S proteasome (10). Under a range of cytotoxic conditions, including hypoxia, nutrient deprivation, pH change, Ca^2+^ depletion from the ER lumen, inhibition of asparagine (N)-linked glycosylation, reduction of disulfide bonds, and overexpression of some proteins, protein malfolding occurs, leading to the accumulation and aggregation of the misfolded proteins in the ER. These abnormalities in the ER are collectively called ER stress. In order to overcome ER stress, the organelle provokes a specific response called UPR (unfolded protein response) (11, 12). The UPR is a signal transduction cascade that limits the accumulation of unfolded proteins, resulting in the reduction of general protein synthesis and the selective activation of the expression of proteins facilitating chaperone activities. The main role of UPR related to the protection of ER can prevent cells from long-term stress, thereby preventing damage to other organelles and the body (Fig. 5A). However, under the conditions of severe ER stress in which cells are unable to adapt to, the UPR triggers the signaling pathway that induces apoptosis (Fig. 5A) (13, 14). It has been reported that Calnexin and Calreticulum belong to a class of chaperones that specifically promote the folding of glycosylated proteins, by interacting with the monoglycosylated glycans present in unfolded glycoproteins and retaining them in the ER until correct folding is achieved (15). Ero1-Lα and PDI (16, 17) can catalyze the formation of disulfide bond, and Ero1-Lα is specifically required for the formation of disulfide bonds in three Lin12-Notch repeats presented in the extracellular domain of Notch (18). The present study revealed that the expression of Ero1-Lα, PDI, and calnexin were upregulated in human GC AGS and BGC-823 cells upon pholidonone incubation. These findings suggest the existence of a mass of unfolded proteins in the ER induced by pholidonone, as the accumulation of misfolded proteins in the ER needs disulfide isomerase to form disulfide bonds to facilitate protein folding, which requires increased amounts of Ero1-Lα, PDI, and Calnexin (Fig. 5A).

Multiple signaling pathways have been identified to be able to initiate cell apoptosis. In this study, we first investigated the effect of pholidonone on the IRE1α-associated pathway. IRE1 is a type I transmembrane protein, with an ER luminal dimerization domain that senses ER stress, and a cytosolic domain containing protein kinase and possessing endonuclease activities (19). It has been proposed that BiP would act as a negative regulator of IRE1α activation (20, 21). The bound of BiP to the luminal domain of IRE1α would maintain IRE1α in an inactive state. The recruitment of BiP, induced by the presence of misfolded proteins in the ER lumen, would prevent BiP from binding with IRE1α, allowing the oligomerization and activation of IRE1α (22, 23). Our present study demonstrated that pholidonone increased the protein expression levels of BiP and IRE1α, indicating that pholidonone is very likely to prevent BiP from binding with IRE1α, allowing the oligomerization and activation of IRE1α (Fig. 5B). Furthermore, we found that the levels of cleaved-Caspase-3 were also elevated. Caspase-3 is a downstream regulator of IRE1α (*via* JNK), which is playing a dominant role in the activation of apoptosis (24, 25). Thus, our findings confirmed that the apoptosis induced by pholidonone was potentially associated with an increased activity of IRE1α-associated pathway, implying the potential antitumor effect of pholidonone.

We further examined the effect of pholidonone on PERK-CHOP signaling pathway. PERK is a type I transmembrane protein with a cytosolic domain with kinase activity, which causes attenuation of translation by phosphorylating the α-subunit of eukaryotic initiation factor-2 (eIF2α) (26–28). Activation of PERK (Fig. 5C) upon ER stress occurs by dimerization and trans-autophosphorylation of its cytosolic domain, leading to the recruitment of its substrate eIF2α, inhibiting the pentameric guanine exchange factor eIF2α from recycling eIF2α to its active GTP-bound form (29). Overexpression of CHOP can lead to cell cycle arrest and apoptosis (30, 31). Expression of CHOP is mainly regulated at the transcriptional level, and it is one of the most highly induced genes during ER stress. Moreover, the PERK/eIF2α signaling pathway plays an essential role in the induction of CHOP (32–34). We found that pholidonone activated PERK and increased the expression of CHOP concomitant with significant apoptosis in AGS and BGC-823 cells. In addition, our data indicated that activation of CHOP *via* PERK/eIF2α/ATF4 signaling could cause the upregulation of pro-apoptotic protein cleaved-Caspase-3, eventually triggering apoptosis (Fig.5C). This finding further confirmed the underlying mechanism of the antitumor effect of pholidonone.

## Conclusion

In recent years, various therapeutic means have contributed to the treatment and prevention of cancers, but these methods can cause irreversible side effects. Thus, the immediate task of the new drug research is to pursue the natural alternatives with high therapeutic properties and low toxicity (35). Pholidonone is a natural antitumor stilbene derivative isolated from EtOAc extract of *P. cantonensis*. Our study indicated that pholidonone displayed antitumor effect by triggering apoptotic cell death of AGS and BGC-823 cells. Moreover, this effect was probably attained by induction of ER stress *via* PERK and IRE1α signaling pathways. In the western blot assay, we detected IRE1α and PERK, which link UPR to autophagy (36); what’s more, the level of cleaved-caspase-3 was also upregulated by pholidonone treatment. These findings suggest that other autophagy and mitochondrial apoptotic pathways may also be involved in the effect of pholidonone on AGS and BGC-823 cells (36, 37), which remains to be discussed in our further study.

To the best of our knowledge, this is the first time that the antitumor mechanism of pholidonone has been revealed. In summary, pholidonone is a small molecule compound with a simple structure, which is prone to be synthesized and modified structurally. All these findings suggest pholidonone may be a promising drug candidate for clinical treatment of cancer in the future.

## Conflict of interest and funding

The authors declare no potential conflicts of interest. This research was financially supported by China Postdoctoral Science Foundation (No. 2016M601865), Natural Science Foundation of Jiangsu Province for Youth (No. BK20170516), Key Talents Project of Yangzhou City (ZDRC20187), and Qinglan Project of Yangzhou University (20180210).
